# Analytical Centrifugal Ultrafiltration as a Tool for High Throughput Process Development in Virus Removal Filtration

**DOI:** 10.1002/biot.70227

**Published:** 2026-04-17

**Authors:** Sebastien Maffeis, Yuliia Fomichova, Levon Manukyan, Albert Mihranyan

**Affiliations:** ^1^ Nanotechnology and Functional Materials Department of Materials Science and Engineering Uppsala University Uppsala Sweden; ^2^ Pharmaceutical Technology Department of Pharmaceutical Biosciences Uppsala University Uppsala Sweden

**Keywords:** centrifugal ultrafiltration, high throughput process development, IgG, nanocellulose‐based filter paper, virus removal filtration

## Abstract

Virus removal filtration constitutes an essential component in biopharmaceutical manufacturing processes for both recombinant and plasma‐derived protein products. The increasing demand for protein‐based therapies necessitates expedited analytical screening methodologies for virus filtration materials and process parameters utilizing high‐throughput development (HTPD) platforms. This study examines a novel HTPD methodology, specifically the implementation of analytical centrifugal ultrafiltration, as a scaled‐down virus removal filtration tool. A nanocellulose‐derived filter membrane of controlled porosity (33 µm thickness, 23 nm) was integrated into a centrifugal ultrafiltration apparatus. The system underwent comprehensive evaluation to determine centrifugal ultrafiltration flux characteristics, product yield across varying buffer viscosities, virus removal capacity utilizing model MS2 (27 nm) and ΦX174 (28 nm) bacteriophages, and IgG throughput capacity. Application of centrifugal force facilitated the generation of multiple filtration pressure gradients suitable for small‐volume sample processing. The resultant flux decay profiles exhibited high reproducibility, demonstrating superior IgG throughput and virus removal efficiency. Overall, the approach adds value to the arsenal of analytical tools useful for HTPD in biopharmaceutics.

## Graphical Abstract and Lay Summary

This work presents a novel high‐throughput development platform integrating nanocellulose‐derived filter membranes into analytical centrifugal ultrafiltration devices for rapid screening of virus removal filtration processes. The results demonstrate the performance of the devices under varying operational conditions, including IgG throughput and model virus clearance at different centrifugal forces, indicating that the proposed scaled‐down methodology enables accelerated screening of virus clearance parameters in biopharmaceutical manufacturing.

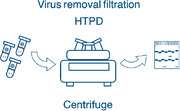

## 1 Introduction

Viral removal filtration constitutes an essential step in biopharmaceutical manufacturing of both recombinant and plasma‐derived proteins. During process development, comprehensive bench‐scale modeling is essential to validate filter throughput capacity and demonstrate effective pathogen elimination from process streams, which can be considerably expensive. Limited understanding of viral clearance mechanisms often leads to filter oversizing and costly process operations. Consequently, there is an increasing demand to expedite the screening of materials and process parameters for virus removal filtration utilizing high‐throughput process development (HTPD) tools.

Currently, optimization experiments for virus clearance on a laboratory scale require substantial volumes, that is, ca. 100 mL feed per trial. Each trial is conducted individually, manually, and over several hours. Under optimum nonfouling conditions, approximately two to four experimental conditions can be completed during a standard 8‐hour workday, depending on the achieved flux and targeted throughput [1]. Virus removal filtration is regarded as a highly complex process wherein a combination of several process variables, including protein concentration, protein hydrophobicity, medium pH, ionic strength, viscosity, additives, pressure fluctuations, and filter chemistry, can influence virus clearance [2, 3], and recently machine learning algorithms have been employed in the attempt to decipher the interplay between variables [4]. Therefore, thorough exploration of numerous process variables inevitably results in extended process validation timeframes, spanning multiple days.

Managing multiple small volume samples containing costly protein products with conventional filter holder devices presents significant practical challenges. Historically, limited attempts have been made to address this requirement. A notable example includes research evaluating a 96‐well virus filter microplate to assess its potential as an innovative micro‐scale HTPD model [1]. In this investigation, we examine an alternative approach, specifically the application of analytical centrifugal ultrafiltration as a tool for scaled‐down virus removal filtration.

Centrifugal devices are widely used as an effective technique for rapid concentration and separation of particles in ultrafiltration/diafiltration (UF/DF) of particles featuring varying sizes at relatively moderate centrifugal forces, contrasting with conventional ultracentrifugation, which is both time‐intensive and requires elevated rotor speeds [5]. Centrifugal filtration's origins trace back to the 1920s, with renewed scientific interest emerging in the 1950s [6]. Researchers reported centrifugal devices incorporating both ultrafiltration membranes [7, 8, 9] and Seitz‐type depth filter pads [10, 11] for safe processing of small‐volume biological fluids, particularly serum. Keup [12] examined the physical principles of centrifugal filtration and identified potential applications in microbiology and aseptic pharmaceutical preparation. A centrifugal filter device equipped with Gradocol (cellulose nitrate) membrane [13] suitable for virus separation was also provided [14].

The use of centrifugal filtration devices in modern times is generally limited to UF/DF for small‐scale purification and separation applications. However, despite the broad availability of tubular centrifugal UF/DF devices (nominal molecular weight cut‐off 3–300 kDa), these systems are not routinely employed in virus removal filter validation protocols because they have a wide pore size distribution. Virus removal filters are distinct from UF/DF membranes, as they possess narrow and highly controlled pore size distribution [15, 16]. For this reason, a dedicated virus filter will show better and more reproducible virus clearance (log_10_ reduction value, LRV >4) than UF/DF membranes featuring nominally smaller pore‐size cut‐off. From HTPD perspective aimed for virus removal filtration validation, analytical centrifugal ultrafiltration devices could present notable advantages. These systems are particularly advantageous when handling limited volumes of protein solutions. The capability to process multiple samples simultaneously using standard laboratory equipment enhances operational efficiency. Additionally, these devices facilitate safe virus‐spiking experiments with minimal infectious material quantities, as their closed falcon tube design prevents sample aerosolization or leakage, thereby ensuring containment integrity.

In this article, we investigate analytical centrifugal ultrafiltration devices equipped with nanocellulose‐based virus removal paper filters, which have demonstrated efficacy in removing both large and small‐size mammalian viruses and bacteriophages [17, 18, 19]. The nanocellulose‐based filter paper exhibits regulated porosity and can be specifically engineered to separate particles of distinct dimensions [20, 21]. This investigation examines the critical operational parameters during centrifugal ultrafiltration for virus removal filtration validation and emphasizes the distinctions from conventional direct‐flow filtration methodologies.

The aim of this work is to explore the feasibility of using analytical centrifugal ultrafiltration devices as potential tools for HTPD in virus filtration and identify distinct benefits/limitations compared to traditional dead‐end filtration under constant pressure conditions.

## 2 Materials and Methods

### 2.1 Materials

#### 2.1.1 Biological Materials and Reagents

Bacteriophages ΦX174 (ATCC 13706‐B1) and MS2 (ATCC 15597‐B1), along with their respective host *Escherichia coli* (Migula) Castellani and Chalmers bacterial strains C (ATCC 13706) and C‐3000 (ATCC 15597), were procured from ATCC (Manassas, VA). BD (Franklin Lakes, NJ) supplied the Agar (214530). Thermo Fisher Scientific provided Tryptone (LP0042B) and yeast extract (LP0021B). Sigma‐Aldrich (St. Louis, MO) supplied phosphate‐buffered saline (PBS; P4417), 2‐mercaptoethanol (M3148), sodium chloride (S7653), NZCYM broth (N3643), and maltose monohydrate (M5885). Bio‐Rad provided any kD Mini‐PROTEAN TGX stain‐free protein gels (4568125), tris/glycine/SDS running buffer (1610732), 4× Laemmli sample buffer (1610747), and Precision Plus Protein unstained protein standards (1610363).

#### 2.1.2 Buffers

Sigma‐Aldrich supplied arginine, histidine, glycine, trehalose, and polysorbate 80. Buffer 1 comprised His/His HCl (20 mM), Glycine (50 mM), and Arginine HCl (50 mM) in deionised water, forming a low viscosity solution. Buffer 2, containing identical components as buffer 1 and additionally 50% (w/v) Trehalose dihydrate and 1% (w/v) polysorbate 80, featured high viscosity. Both buffers were adjusted to pH 5. The pH and components of buffer 1 and 2, including amino acids, sugars, and surfactants, were mimicking buffers compositions for the final formulation of therapeutic IgG products.

#### 2.1.3 Immunoglobulin G

CSL Behring generously provided polyclonal plasma‐derived immunoglobulin G (IgG) as a post‐chromatography intermediate process solution (11 mg mL^−1^, pH 4.9).

### 2.2 Filter Preparation

The filters were fabricated utilizing Cladophora cellulose (FMC BioPolymer G‐3095‐10 batch, USA) suspended in deionized water at a concentration of 0.1 wt %. The suspension underwent high‐pressure homogenization (LM20, Microfluidics, USA) by passing through chambers of 200 µm (two passes) and 100 µm at 1800 bar. Then, the homogenized cellulose suspension was vacuum‐filtered through a membrane (Durapore, 0.65 µm DVPP, Merck Millipore, Burlington, MA). The resultant wet‐cake was thermally processed at 80°C using a hot press (Carver, USA), yielding paper sheets with a thickness of 33 µm. The structure and performance of these filters in conventional direct flow filtration mode has been covered previously in applications including bioprocessing [17, 18, 19, 21, 22] as well as water purification [23, 24] and, therefore, will be outside of the scope of the present article.

### 2.3 Thermoporometry

Differential scanning calorimetry (Mettler Toledo DSC3) instrument was used for thermoporometry. Filter paper samples (1.5–2 mg) were soaked into deionized water overnight at room temperature. Water was decanted, and the samples were placed in aluminum crucibles with a lid. Samples were cooled down to 248.15 K (−25°C) at a rate of 10 K min^−1^ followed by heating to 277.15 K (4°C) at a rate of 0.7 K min^−1^.

The pore size was calculated according to Landry:

(1) $$\;r_{p}=\frac{-19.082}{\Delta T+0.1207}+1.12$$

where *r_p_* is the radius of pore (nm) and *ΔT* is the difference between the peak maximum for melting of pore‐confined water and peak value for melting of bulk water, experimentally determined at 0.6 ± 0.01°C.

### 2.4 Centrifugal Filter Device

**FIGURE 1:**
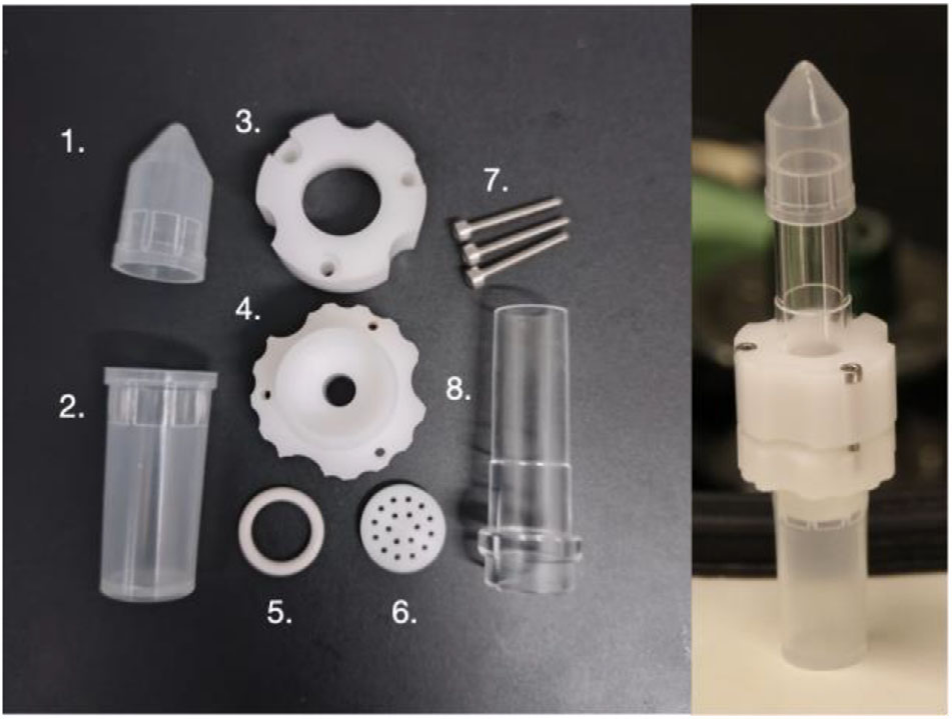
Illustration of analytical centrifugal ultrafiltration device: (1) feed cap, (2) permeate cap, (3) top holder, (4) bottom holder, (5) O‐ring, (6) mesh, (7) screw threads, and (8) feed compartment tube.

Different designs of centrifugal filter holder have been implemented in different materials (e.g., stainless steel, glass) previously, for example, by Keup [12] or Boerner [10]. Therefore, the design of the presented device for HTPD shall be considered only illustrative. Figure 1 depicts the design of the centrifugal filter holder utilized in this study. The experimental setup employed a device consisting of detachable feed (1) and permeate (2) caps, a two‐piece holder (3, 4), O‐ring (5), perforated mesh disc (6), three screws threads (7), and a feed compartment tube (8). The filter paper sheet was positioned on the perforated disc (5) and secured with an O‐ring (5) between the holder's top and bottom components (3, 4), fitted with a plastic tube (8). Three screw threads (7) penetrated both holder pieces (3, 4) to ensure firm assembly, preventing torsional stress on the filter paper during installation and potential structural compromise. Transparent plastic parts (1, 2, 8), attached to both ends of the holder device, facilitated visual monitoring of the filtration process. The centrifugal force generated the necessary transmembrane pressure that drives the filtration. The holder's plastic components underwent chemical sterilization using a solution of 25% ethanol and 5% formalin. Prior to centrifugation, the assembled device containing the sample was positioned inside a 50 mL falcon tube, to prevent inadvertent splashing. For operation, an aliquot of a sample was placed in the upper feed compartment tube (8) of the device and then centrifuged. Figure S1 in Appendix presents a blueprint of presented device.

### 2.5 Centrifugal Ultrafiltration Procedure

A Sorvall ST16 centrifuge (Thermo Scientific) was operated at multiple rotational velocities, that is, 2000, 3000, 4000, and 5000 rpm. Prior to experimentation, the filter holder mass was recorded, including that of detachable permeate (bottom) cap, followed by the addition of 2 mL sample volume. To evaluate filtration kinetics, virus removal efficiency, and protein flux as a function of centrifugal speed, the mass of the filtered sample was monitored gravimetrically at predetermined intervals. In the current experimental set‐up, up to 16 samples could be run in parallel at a pre‐defined rotational velocity.

The pressure differential during centrifugal filtration was determined using the following equation:

(2) $$\Delta P=\frac{m\cdot g\cdot G}{A}\;$$

where ΔP represents the pressure differential (N m^−^^2^), m denotes mass (kg), g signifies gravitational acceleration (9.8 m s^−^^2^), G indicates relative centrifugal force (RCF or G‐force), and A represents the filter disc surface area (m^2^). The RCF calculation follows:

(3) $$G=1.118\cdot 10^{5}\cdot r\cdot R^{2}$$

where r represents the rotor radius, measured as the distance (cm) from the rotor center to the filter surface (10 cm in this setup), R is the rotor speed (rpm). The theoretical basis of centrifugal of filtration is covered in detail in Appendix.

In the current experimental set‐up, up to 16 samples could be run in parallel at pre‐defined rotational velocity. No validation of the assumption of worst‐case conditions compliant with definitions of ICH Q5A(R2) was conducted at this time.

### 2.6 Virus Retention Test

The virus clearance capability of the centrifugal devices was estimated following in general the previously described protocols [18, 21, 24] as described in detail below.

#### 2.6.1 MS2 Coliphage PFU Titration

The MS2 bacteriophage stock was prepared by propagation in *E. coli* (Migula) Castellani and Chalmers strain C‐3000 host bacteria at exponential growth phase in NZCYM broth (2.2% NZCYM and 0.2% maltose in deionized water), followed by centrifugation for clarification and subsequent 0.2 µm filtration. Bacteriophage titer was determined by the double layer agar plaque assay. The bacteriophage stock underwent serial 10‐fold incremental dilution and subsequent inoculation into *E. coli* host strain at exponential growth phase. Then, 100 µL of bacteriophage suspension was combined with 200 µL of  *E. coli* host strain culture and 1 mL of soft agar (containing 0.4% agar, 2.2% NZCYM, and 0.2% maltose in deionized water). This mixture was then distributed onto prepared ventilated hard agar plates (1% agar, 2.2% NZCYM, and 0.2% maltose in deionized water) and incubated at 37°C for 7 h. Each dilution required duplicate plating for enhanced accuracy, with plaque‐forming units (PFU) mL^−1^ calculation following the formula:

(4) $$log_{10}\left(\frac{PFU}{mL}\right)=log_{10}\left(\frac{N}{0.1\;\times \;DF}\right)$$

where N represents the plaque count, 0.1 denotes the virus volume (mL) introduced, and DF indicates the dilution factor employed.

To determine the bacteriophage titer after filtration, the MS2 phage feed samples underwent serial dilution up to 1:10^6^ using NZCYM broth. The permeate samples remained undiluted. The *E. coli* (Migula) Castellani and Chalmers strain C‐3000 host strain was cultured in NZCYM broth at 37°C for 3 h in a shaking incubator (INCU‐Line ILS 4) with 220 rpm agitation to achieve the exponential growth phase. Bacteriophage titer was determined by the double layer agar plaque assay methodology as detailed above.

#### 2.6.2 ΦX174 Bacteriophage PFU Titration

The ΦΧ174 bacteriophage, a small (28 nm) icosahedral nontailed virus, stock was propagated through inoculation of *E. coli* host bacteria strain at exponential growth phase in Luria‐Bertani broth (LB) (containing 1% tryptone‐t broth, 0.5% yeast extract, 1% NaCl in deionized water). Following 5 h of incubation at 35°C with 120 rpm agitation, bacteriophage was harvested and clarified by centrifugation at 5000 g for 10 min. The resultant suspension was stored at 4°C until use. Bacteriophage titer was determined by the double layer agar plaque assay as detailed above.

#### 2.6.3 Bacteriophage Filtration

The filters were autoclaved prior to bacteriophage filtration trials; no filter pre‐wetting was performed. The experimental protocol involved spiking IgG (11 mg mL^−1^) in PBS (pH 7.4) or in buffer 1 solution with model bacteriophage, that is, ΦΧ174 and MS2, respectively, to achieve an approximate final titer of 10^6^ PFU mL^−1^. The filtration process utilized a centrifugal ultrafiltration device, wherein 2 mL of feed solution was introduced into the holder apparatus positioned within a 50 mL centrifuge tube. Subsequently, the tube underwent centrifugation at 2000–5000 rpm, with filtration endpoint determined through visual assessment.

The collected permeate samples were maintained at 4°C pending PFU analysis. Bacteriophage removal efficiency was quantified using log reduction values (LRV), calculated as:

(5) $$\text{LRV}=\log_{10}\;{\text{PFU}}_{\mathrm{mL}}\left(\text{feed}\right)-\log_{10}\;{\text{PFU}}_{\mathrm{mL}}\left(\text{permeate}\right)$$

The detection threshold, specifically ≤0.7 PFU mL^−1^, of the PFU assay under the implemented experimental parameters corresponds to ≤5 bacteriophages mL^−1^, which manifests as a single observable plaque in one of the duplicate plates for nondiluted samples. Virus clearance corresponding to LRV≥4 (i.e., ≥99.99%) was used as a threshold for robust removal [25].

### 2.7 Protein Recovery

#### 2.7.1 SEC‐HPLC Analysis

A Hitachi Chromaster HPLC system (Hitachi High‐Tech, Japan) equipped with a 5110 Pump (operating flow rate range 0.001 to 9.999 mL/min), Auto Sampler 5260 (Withstand pressure 60 MPa, Loop injection method), and a 6310 Column Oven, vertical type was used. Detection was performed using a Chromaster 5420 UV‐VIS spectrophotometric detector at 280 nm wavelength. Separation was realized by size exclusion chromatography (SEC) on a TSKgel G4000 SWXL column (300 × 7.8 mm, 8 µm) with TSKgel SWXL guard column (6.0×40 mm, 7 µm). Mobile phase, consisting of 0.1 M phosphate buffer (pH 6.7) with 0.1 M sodium sulphate (Na_2_SO_4_) and 0.05% sodium azide (NaN_3_), was eluted isocraticly at 0.5 mL/min flow rate. The injection volume was 10 µL, and the working temperature was 22°C. The working pressure was around 51 bar, and the run time was set at 45 min. The samples were pre‐filtered through 0.45 µm filter prior to analysis to remove potential aggregates.

A calibration curve for the quantification of IgG was constructed as follows. Four working concentrations were prepared from a stock solution of 11 mg/mL by dilution with a PBS buffer at pH 6.8. The calibration curve was constructed by plotting the peak area under the curve (AUC) of ​​each standard as a linear function of its concentration (r^2^ = 0.999).

#### 2.7.2 SDS‐PAGE Analysis

Specimens were mixed with Laemmli buffer supplemented with 5% (v/v) 2‐ME, followed by thermal treatment at 100°C for 10 min. Protein separation was achieved using a precast separating gel at 270 V in a Mini‐PROTEAN Tetra Vertical Electrophoresis Cell (Bio‐Rad). Gel documentation was performed using the Gel Doc EZ System (Bio‐Rad). Band intensity quantification was conducted using Image Lab 4.0.1 analysis software (BioRad).

#### 2.7.3 Total Protein Content Biuret Assay

For protein recovery quantification, specimens were combined with total protein biuret reagent (Merck) maintaining a 1:3 volumetric ratio. Following a 10‐minute incubation period, absorbance measurements were obtained at 540 nm utilizing a Tecan M200 microplate reader.

### 2.8 Statistical Analysis

Descriptive statistical analysis was used to obtain mean, standard deviation of the mean, median, and range in Python 3.13 using pandas from *numpy* (numerical Python) library and statistical submodule *scipy* (scientific Python). Considering the small population size (n = 6‐7), t‐value from the Student's distribution was used to calculate 95% confidence interval (CI) and compared to z‐score = 1.96, which is typically used for normally distributed larger population sizes (n = 30) as follows:

(6) $$\;CI_{95\%\left(low\right)}=\mu -SE\cdot t_{value}$$

(7) $$\;CI_{95\%\left(high\right)}=\mu +SE\cdot t_{value}$$

where μ is the mean of non‐zero values n*, SE is the standard error of mean, and t_value_ is the t‐critical value.

## 3 Results

In biopharmaceutical applications, filtration typically operates either under constant pressure or constant flow conditions. However, centrifugal filtration is different, as it operates under constant G‐force (or constant relative centrifugal force, RCF), determined by rotor speed, sample mass, and rotor radius. The latter distinguishes centrifugal filtration from conventional methods, as neither pressure nor flow remains constant during operation. It should be noted that the topic of filtration under variable pressure and variable flow has received limited attention in biopharmaceutical processing, especially for analytical process development applications, although some limited applications have been described, for example, for cake filtration with a centrifugal pump to assess process capacity and fouling behavior [26]. ICH Q5A(R2) Guideline states that pressure/flow interruptions and fluctuations are potentially critical parameters in virus filtration [27]. It should however be mentioned that studying the effects of pressure/flux interruptions during analytical centrifugal filtration was not in the scope of this study, although varying pressure and flux decline may indirectly mimic worst‐case operation conditions.

**FIGURE 2:**
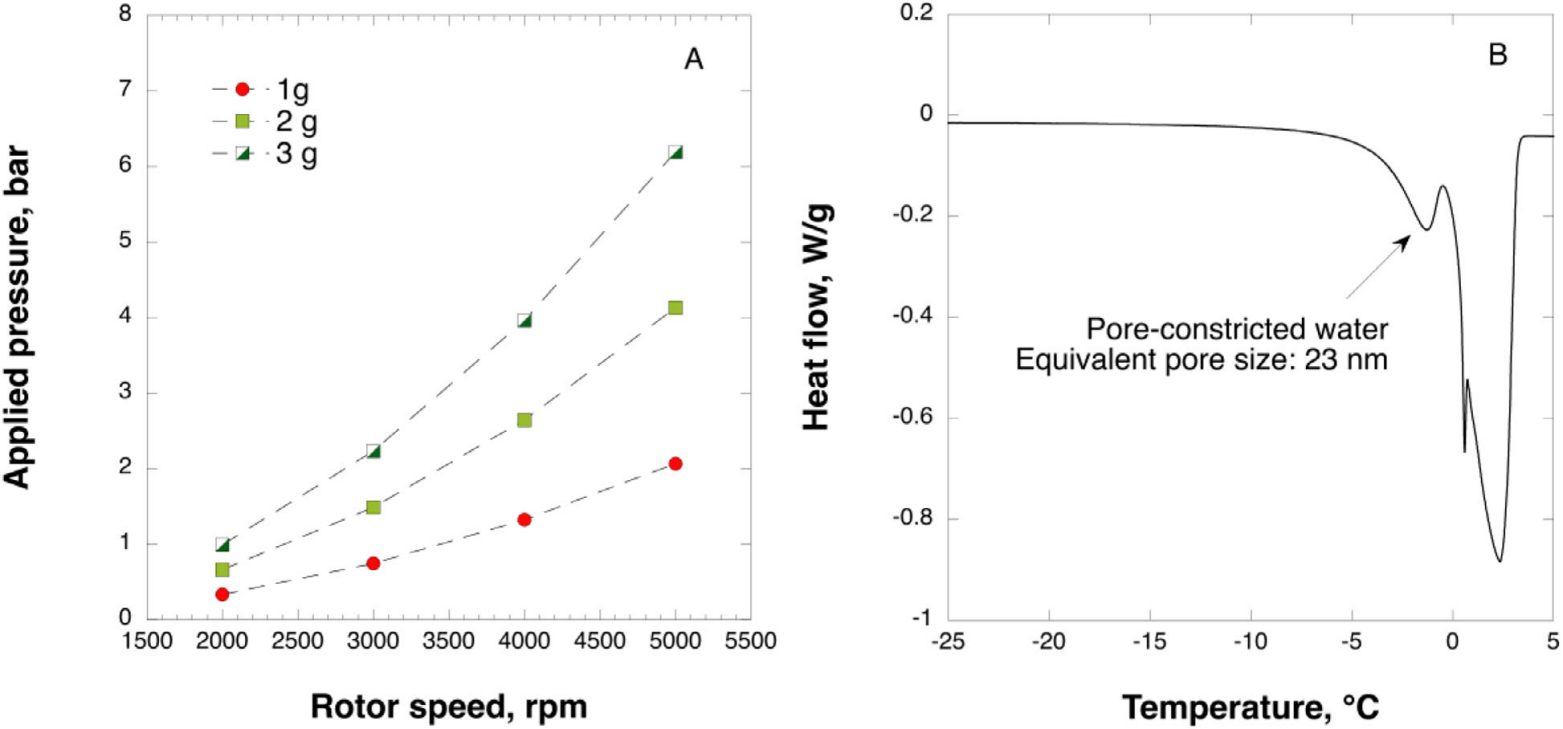
Dependence of applied pressure on rotor speed (A) and thermoporometry differential scanning calorimetry scan of nanocellulose‐based filter paper pre‐soaked in water (B).

Figure 2A demonstrates the relationship between the sample mass, rotor speed, and applied pressure. The sample sizes of 1, 2, and 3 mL correspond to a volumetric load of 7.5, 15, and 22.5 L/m^2^. Increasing sample mass and rotor speed result in higher applied pressure, which changes in nonlinear fashion. For virus removal applications, filtration pressure rarely exceeds 3 bar. Therefore, the centrifugal ultrafiltration configuration shown in this work presents a viable approach for simulating filtration scenarios using minimal volumes (1‐3 mL) and when operated at moderate centrifugation speeds (≤5000 rpm). In this study, the centrifugal ultrafiltration device was fitted with a 33 µm in thickness nanocellulose‐based filter paper. Figure 2B shows the thermoporometry analysis data, depicting the heat flow curve of ice (water) melting inside the pores of the nanocellulose‐based filter paper. From the melting point of water constricted inside the pores, a corresponding pore size of 23 nm was obtained, which is in the desired range for separation of model small‐size viruses (27‐28 nm) from IgG (12 nm).

### 3.1 Effect of Buffer Viscosity on the Rate of Analytical Centrifugal Ultrafiltration

**FIGURE 3:**
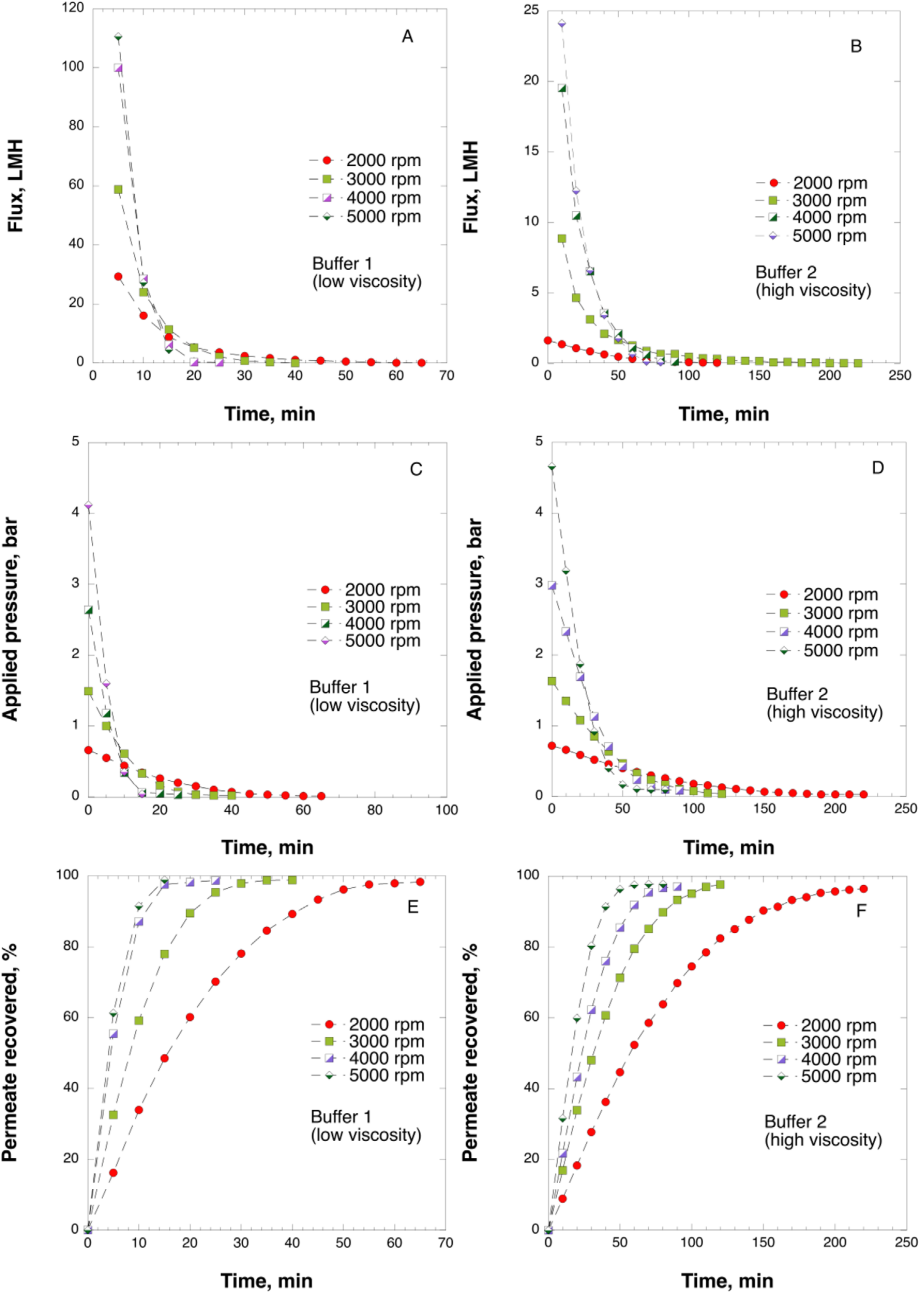
A‐B. Rate of centrifugal ultrafiltration versus time for buffers of varying viscosity; C‐D. Applied pressure due to centrifugal force versus time for buffers of varying viscosity; E‐F. Permeate recovery as a function of centrifugation time for buffers 1 and 2.

Figure 3A,B illustrates the relationship between filtration flux and time for buffers with different viscosities at various rotor speeds. The data demonstrates that both rotor speed and buffer viscosity significantly influence the filtration flux. The graphs indicate a more pronounced decrease in flux at higher rotor speeds, correlating with the rapid decline in transmembrane pressure. Figure 3C,D presents the pressure reduction dynamics during centrifugal ultrafiltration through nanocellulose‐based filter paper of buffers with varying viscosity. The flux decline region exhibits a steeper gradient for samples processed at higher rotor speeds compared to those at lower speeds. Figure 3D reveals that buffer 2, characterized by high viscosity, demonstrates a slower pressure reduction rate compared to buffer 1. Consequently, although pressure is maintained over the filter face for an extended duration, the filtration process in buffer 2 requires significantly more time than in buffer 1. Figure 3E,F depicts the temporal evolution of permeate recovery for buffers 1 and 2. The graph reveals nonlinear recovery behavior. For instance, during the centrifugal ultrafiltration of buffer 2, recovering the initial 50% of the product requires approximately 20–70 min, depending on rotor speed, while the remaining 50% necessitates a threefold longer duration. Indeed, this nonlinear recovery pattern may present challenges in parallel sample analysis under varying process conditions during HTPD. Therefore, filtration behavior investigations may be expedited by focusing solely on the linear portion of the curve, up to 50% product recovery. Conversely, product recovery analysis in the nonlinear region may prove valuable when investigating worst‐case scenarios associated with rapid flux reduction, such as simulating filter fouling or virus breakthrough at low transmembrane pressures. Collectively, Figure 3A‐F demonstrate the dynamic characteristics of centrifugal filtration and its sensitivity to operational parameters such as rotor speed and buffer viscosity. It should be noted that up to 16 samples could be tested per run under the experimental conditions. Considering that the typical test takes between 20 and 240 min/run, depending on the process parameters and product viscosity as discussed above, potentially hundreds of samples can be readily processed per day using small volume samples, which starkly contrasts 2–4 tests/day that can be done using the standard filtration set up and large product volume, see further discussion in Section 3.4.

### 3.2 Effect of Analytical Centrifugal Ultrafiltration on LRV

**FIGURE 4:**
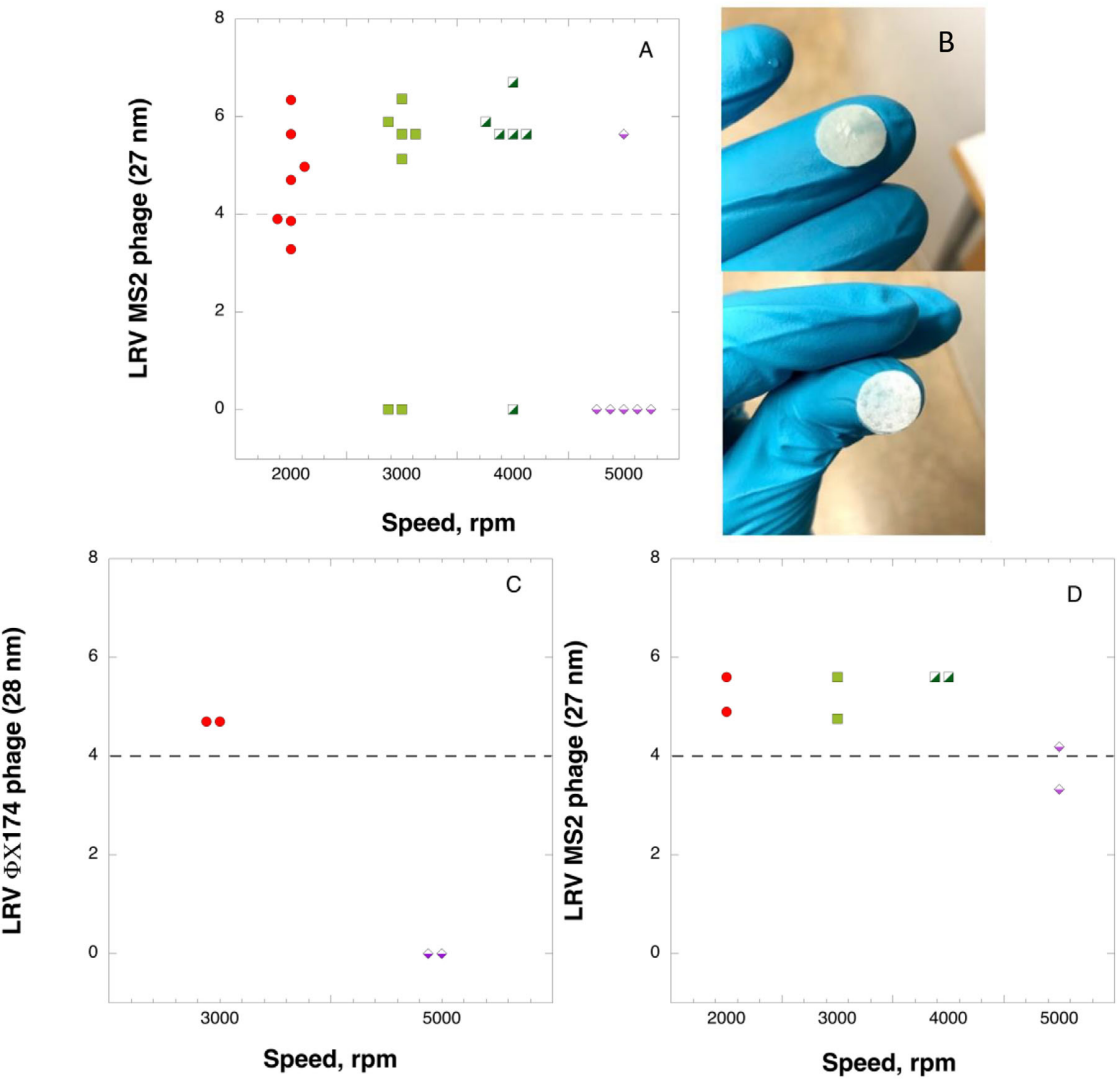
A. MS2 phage clearance at varying rotor speeds; B. Images of filter surface after centrifugal ultrafiltration at 5000 rpm, including nanocellulose‐based filter paper (upper panel) and support paper (lower panel); C. ΦX174 phage clearance at varying rotor speeds; D. LRV MS2 phage clearance to half‐volume at varying rotor speeds. Dotted line represents 4 log_10_ virus clearance threshold. Dotted line represents 4 log_10_ virus clearance threshold.

Figure 4 demonstrates the effectiveness of centrifugal ultrafiltration devices in evaluating virus clearance capacity. Statistical analysis data of observed LRVs is further presented in Table 1. It should be noted that the current setup features a volumetric load of 15 L/m^2^ load (2 mL), which is lower than recommended ≥ 50 L/m^2^ load for virus clearance studies by Parenteral Drug Association's Technical Report 41 [28]. However, considering that the purpose of HTPD is screening rather than full‐scale validation, the relatively low load is acceptable and the obtained results indicative of overall filter behavior under challenging conditions of variable pressure and flux. The data indicate that the nanocellulose‐based filter paper achieves greater than 4 log_10_ virus clearance for the majority of samples when operated at rotor speeds below 5000 rpm (mean_2000_ = 4.67; median_2000_ = 4.70; mean_3000_ = 4.09; median_3000_ = 5.64; mean_4000_ = 4.92; median_4000_ = 5.64, see Table 1). However, at 5000 rpm, the elevated pressure during centrifugal ultrafiltration typically induces filter failure, resulting in complete virus penetration (mean_5000_ = 0.94; median_5000_ = 0, see Table 1). Specifically, only one out of six samples demonstrated log_10_ virus clearance exceeding 4 for MS2 phage. As illustrated in Figure 4B, the virus breakthrough observed at 5000 rpm results from physical damage of the filter, evidenced by visible dents and cracks in both the active nanocellulose‐based filter and support paper. The virus breakthrough at 5000 rpm was additionally verified in auxiliary ΦΧ174 phage experiments, as shown in Figure 4C. Furthermore, experiments on MS2 phage clearance conducted at half‐volume filtration demonstrated enhanced virus clearance efficiency at 5000 rpm compared to full‐volume filtration, as illustrated in Figure 4D. This observation suggests that filter damage occurs predominantly during the nonlinear filtration phase. Additional research is required to fully understand the filtration dynamics in both linear and nonlinear regions. In contrast, centrifugation at 2000, 3000, and 4000 rpm produced no visible structural damage. In this context, it is important to mention that virus breakthrough was shown to occasionally occur in samples centrifuged at 3000 and 4000 rpm, without visible structural damage. In the cases where no visible damage could be ascertained, the virus leakage in these samples was attributed to inherent variability in the filter structure, further validating the method's sensitivity in process development applications.

**TABLE 1:** Statistical analysis of LRV MS2 phage through an analytical centrifugal ultrafiltration device.

RPM	n	n^*^	Mean	Mean^*^	SD	Median	Range	95% CI^*^
2000	7	7	4.67	4.67	1.08	4.70	3.28‐6.34	3.67‐5.67
3000	7	5	4.09	5.73	2.61	5.64	0.00‐6.36	5.18‐6.29
4000	6	5	4.92	5.90	2.23	5.64	0.00‐6.70	5.33‐6.47
5000	6	1	0.94	5.64	2.10	0.00	0.00‐5.64	—

* Non‐zero data points;.

RPM 2000: n* = 7 (df = 6), t‐critical = 2.447 (vs. z‐score = 1.96)

RPM 3000 and RPM 4000: n* = 5 (df = 4), t‐critical = 2.776 (vs. z‐score = 1.96)

### 3.3 Effect of Analytical Centrifugal Ultrafiltration on IgG Throughput

**FIGURE 5:**
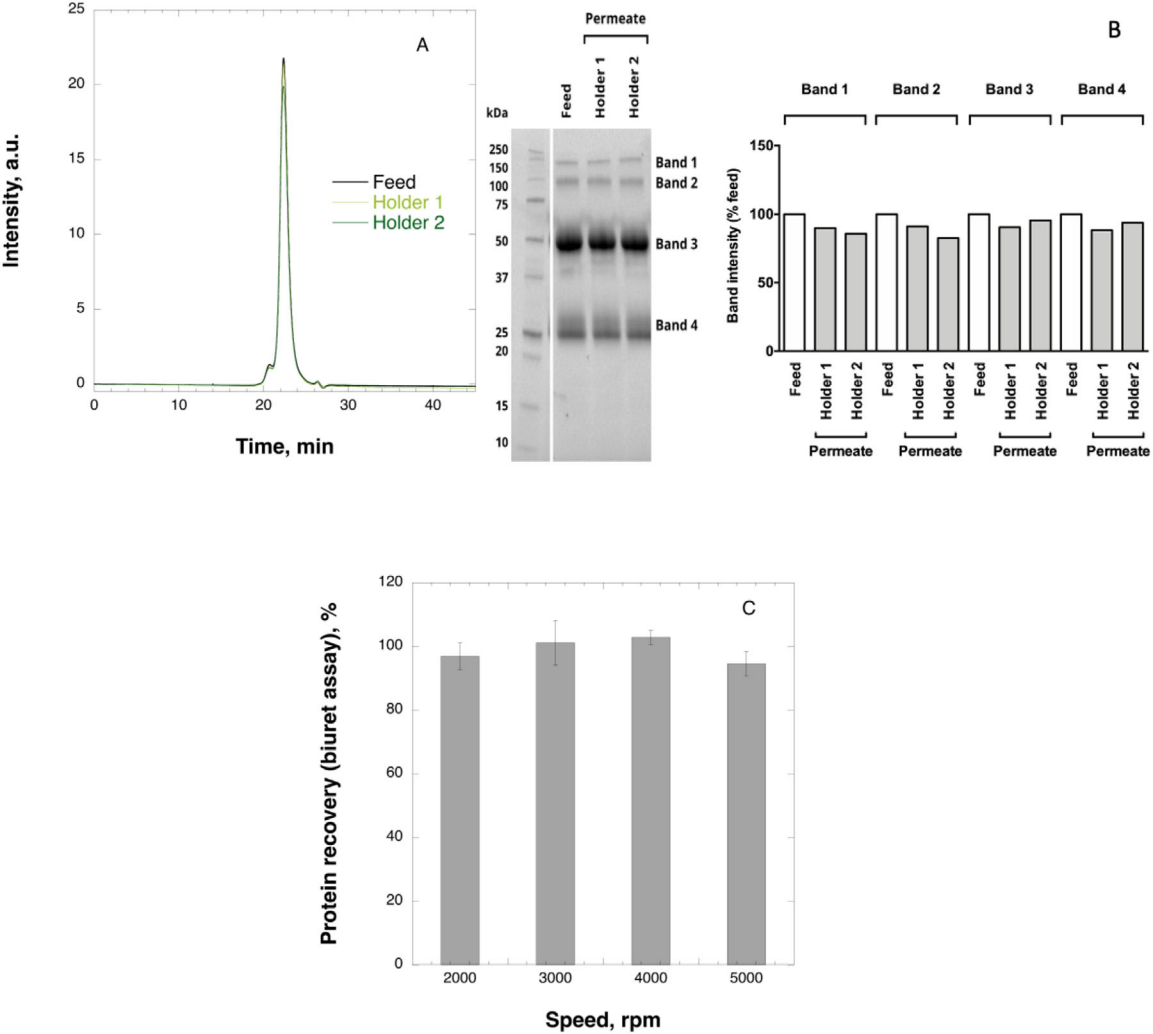
Illustrative SEC‐HPLC (A) and SDS‐PAGE (B) analysis of IgG (11 mg mL^−1^) feed and permeate samples following centrifugal ultrafiltration at 3000 rpm; Total protein biuret assay (C) recovery of IgG (11 mg mL^−1^) following centrifugal ultrafiltration using 33 µm virus removal filter paper.

The efficiency of IgG (11 mg mL^−1^) recovery during centrifugal ultrafiltration was evaluated across various rotor speeds with complementary analytical methods. Figure 5A,B presents illustrative SEC‐HPLC and SDS‐PAGE analysis results at 3000 rpm, confirming high IgG yield and demonstrating that distinct protein fractions remained unaltered by centrifugal filtration. The latter is illustrated by examination of IgG monomer peak in Figure 5A and Bands 3 and 4 in Figure 4B, corresponding to IgG heavy and light chains, respectively. It is seen in these plots that no IgG aggregation or fragmentation is observed in the permeate samples. The SEC‐HPLC recovery data at varying rotor speeds (summarized in Appendix Table S2) is further corroborated in Figure 5C, showing IgG recovery quantified by total protein biuret assay. The results demonstrated consistently high protein recovery rates, averaging above 95% across all experimental conditions.

### 3.4 Commentary on Application

As discussed above, with the current set up it is possible to test a large number of small samples (potentially hundreds per day) simultaneously. This is a conservative estimate, as the number of samples that can be run in parallel is largely dependent on the capacity of the bench‐top laboratory centrifuge. In this report, the bench‐top centrifuge allows a maximum of 16 devices placed in 50 mL Falcon tubes to be tested. However, modern high‐capacity bench‐top laboratory centrifuge models may run up to 40× 50 mL Falcon tubes in parallel. Furthermore, the effective filtration area per device and volumetric load (L/m^2^) per experiment can further be adjusted, suggesting that devices could be fitted inside even smaller tubes, dramatically increasing the number of individual samples that can be tested (e.g., 96× 15 mL tubes). Whilst the total number of runs per day will depend on the viscosity of the medium and typical operational rotor speed, it will remain sufficiently high to substantially expedite and intensify process development. Also, while the recommended upper limit for the presented nanocellulose‐based paper filter is below (≤4000 rpm), higher rotor speeds may be possible for membranes made of different chemistry or featuring higher wet strength. The latter requires further investigations.

It is further relevant to compare analytical centrifugal ultrafiltration with traditional filter plate technologies for HTPD. Filter plate technologies have been implemented for screening membrane chromatography platforms [29], filter throughput studies [30], and virus removal filtration [1]. The HTPD microplate method requires a liquid handling robot equipped with a pressure control unit and a custom‐made microplate holder. The method described by Tang et al. [1] relies on specialized liquid level detection capabilities of pipetting robot that may be available only on select instruments. Compared to the filter plate method, analytical centrifugal ultrafiltration uses a common bench‐top centrifuge, allowing independent monitoring of flux and mass transfer for each device/membrane/process condition in a straightforward and cost‐efficient manner. The main conceptual difference is the mode of operation: the microplate method operates under constant pressure, whereas the centrifugal method operates under variable flux and pressure conditions. Further research is needed to benchmark both methods for industrial and research applications.

## 4 Conclusions

This article investigated the feasibility of using centrifugal ultrafiltration for virus removal studies. In this context, centrifugal ultrafiltration presents significant advantages for HTPD. The application of centrifugal force enables the generation of diverse filtration pressure gradients necessary for processing small‐volume samples. Moreover, the system allows simultaneous processing of numerous samples at a specified centrifugal force, making these devices particularly valuable for HTPD applications, offering more data in a shorter time. The observed flux decay curves demonstrated excellent reproducibility, featuring high IgG throughput and virus clearance capacity. The scale‐down model effectively predicted filter performance across varying applied pressures. A key advantage of this system lies in its compatibility with standard laboratory centrifuges. The method further features built‐in “worst‐case” stress test not easily achieved by alternative systems. Regarding limitations of the presented approach, one should carefully evaluate the dynamic behavior of centrifugal ultrafiltration under fluctuating flux and pressure parameters, although these conditions may be useful to simulate worst‐case scenarios. Another limitation lies in the small volumetric load per surface area, which is, however, offset by the screening character of the procedure and several other benefits. In general, the topic of virus filtration under conditions of variable pressure and flux is an interesting and still largely underexplored area for continued studies. Future efforts should be directed to further validating the filter devices for different types of filter chemistries and process variables as well as emerging types of biologicals, such as viral vectors and extracellular vesicles (exosomes).

## Author Contributions

Conceptualization, A.M. and L.M.; methodology, A.M., L.M.; formal analysis, S.M., Y.F., L.M.; investigation, S.M., Y.F., L.M.; resources, A.M.; data curation, L.M. and A.M.; writing – original draft preparation, A.M.; writing – review and editing, S.M., Y.F., L.M., A.M.; visualization, L.M., A.M.; supervision, A.M.; project administration, A.M.; funding acquisition, A.M. All authors have read and agreed to the published version of the manuscript.

## Funding Information

This work was in part funded by the EIT Health Innovation by Idea project VIREPAP (19104). EIT Health is supported by the European Institute of Innovation and Technology (EIT), a body of the European Union.

## Conflicts of Interest

The corresponding author (A.M.) is the inventor behind the IP pertaining to virus removal filter paper.

## Supporting information




**Supporting File**: biot70227‐sup‐0001‐Appendix.docx.

## Data Availability

Data will be available upon reasonable request from authors.
