# Supplementary material for: Analytical Centrifugal Ultrafiltration as a Tool for High Throughput Process Development in Virus Removal Filtration
Source: Biotechnol J. 2026 Apr 17;21:e70227. doi: 10.1002/biot.70227 (PMC13088221; doi:10.1002/biot.70227)
Supplement: Supplementary file 1 — Supporting File: biot70227‐sup‐0001‐Appendix.docx. [file BIOT-21-e70227-s001.docx]

**Appendix**

*Theory*

The physical principles governing pressure centrifugal filtration have been described in the literature previously and are well known.[Keup Experientia (1954); 10: 389-391 https://doi.org/10.1007/BF02160557] The centrifugal pressure exerted of membrane surface was calculated from the centrifuged mass *m*, the mean radius of the orbit of the centrifuged mass *r*, the angular velocity *w*, and A membrane surface area:

$\Delta P=\frac{m\cdot r\cdot w^{2}}{A}$ (1)

The angular velocity depends on the time *t* required for one revolution:

$w=\frac{2\pi\cdot RPM}{t}$ (2)

Substituting this relationship into the centrifugal force equation yields:

$\Delta P=\frac{m\cdot r\cdot4\pi^{2}\cdot{RPM}^{2}}{A\cdot t^{2}}$ (3)

wherein r is the radial distance in centimeters, RPM is the rotational speed in revolutions per minute

Expression 3 can be converted to the more commonly used RPM-based formula by recognizing that t = 60/RPM, which leads to the expression involving relative centrifugal force (RCF):

$RCF=\frac{w^{2}\cdot r}{g}=1.118\cdot{10}^{-5}\cdot{r\cdot RPM}^{2}$ (4)

wherein g is the standard gravitational acceleration equal to 981 cm/s^2^. The constant 1.118 × 10⁻⁵ derives from the unit conversions based on $\frac{(2\pi/60)^{2}}{981}$. Expression 1 is also commonly stated in mathematically equivalent terms as follows:

$\Delta P=\frac{m\cdot g\cdot RCF}{A}=\frac{m\cdot g\cdot\left( \frac{w^{2}\cdot r}{g} \right)}{A}=\frac{m\cdot r\cdot w^{2}}{A}$ (5).

Figure S1. Filter holder device blueprint.

Table S1. Summary of IgG recovery (%) through analytical centrifugal ultrafiltration device.

| **Rotor speed, rpm** | **Sample** | **Intensity AUC, mAU** | **Concentration, mg mL^-1^** | **Recovery, %** |
| --- | --- | --- | --- | --- |
| - | Feed | 1557.92 | 10.58 | - |
| 2000 | Holder 1 | 1518.52 | 10.30 | 97 |
| 2000 | Holder 2 | 1398.38 | 9.48 | 90 |
| 3000 | Holder 1 | 1594.16 | 10.82 | 102 |
| 3000 | Holder 2 | 1545.42 | 10.48 | 99 |
| 4000 | Holder 1 | 1539.36 | 10.44 | 99 |
| 4000 | Holder 2 | 1530.97 | 10.38 | 98 |
| 5000 | Holder 1 | Membrane damage | - | - |
| 5000 | Holder 2 | 1532.59 | 10.4 | 98 |
